# Giant splenic artery aneurysm: case report

**DOI:** 10.1590/1677-5449.20230108

**Published:** 2023-11-27

**Authors:** Aleksey Vasilyevich Shabunin, Vladimir Vladimirovich Bedin, Mikhail Mikhailovich Tavobilov, Аleksey Andreevich Karpov, Fariza Fayzulloevna Alieva

**Affiliations:** 1 Botkin Hospital, Moscow, Russia.; 2 Russian Medical Academy of Continuous Professional Education – RMACPE, Moscow, Russia.

**Keywords:** giant splenic artery aneurysm, aneurysm management, aneurysm resection, splenectomy, distal pancreatic resection, splenic artery intervention, vascular surgery, aneurisma gigante da artéria esplênica, manejo de aneurismas, ressecção de aneurisma, esplenectomia, pancreatectomia distal, intervenção na artéria esplênica, cirurgia vascular

## Abstract

True splenic artery aneurysms are exceedingly rare and the medical literature contains only a limited number of reports on this pathology. Presently, there remains a lack of consensus regarding the optimal management and treatment approaches for patients in this category. Over the course of the last century, significant changes have occurred in the realm of surgical options, transitioning from open and endovascular procedures to the more advanced laparoscopic and robotic interventions. The propensity for these aneurysms to rupture underscores the need for timely intervention. The risk of rupture is notably elevated in patients harboring giant splenic artery aneurysms. In this report, we present the case of a 55-year-old woman diagnosed with a giant splenic artery aneurysm measuring 12x12 cm in diameter. She presented with notable weakness, discomfort, and pain in the left subcostal area. In response to her complaints and after thorough evaluation, we opted for a surgical procedure encompassing distal pancreatic resection in conjunction with splenectomy and resection of the giant splenic artery aneurysm.

## Resumo

Os aneurismas verdadeiros da artéria esplênica são extremamente raros, e há um número limitado de relatos sobre essa condição na literatura médica. Atualmente, não há consenso sobre as abordagens ideais de manejo e tratamento para pacientes que se enquadram nessa categoria. Ao longo do século passado, ocorreram mudanças significativas no domínio das opções cirúrgicas, passando de procedimentos abertos e endovasculares para intervenções laparoscópicas e robóticas mais avançadas. A propensão à ruptura do aneurisma ressalta a necessidade de intervenção em tempo oportuno. O risco de ruptura é notavelmente elevado em pacientes com aneurismas gigantes da artéria esplênica. Neste relato, apresentamos o caso de uma mulher de 55 anos diagnosticada com aneurisma gigante de artéria esplênica medindo 12x12 cm de diâmetro. A paciente apresentava fraqueza notável, desconforto e dor na região subcostal esquerda. Em resposta às suas queixas e após avaliação minuciosa, optamos por um procedimento cirúrgico que incluiu pancreatectomia distal associada a esplenectomia e ressecção do aneurisma gigante da artéria esplênica.

## INTRODUCTION

True splenic artery aneurysms were initially described by M. Beaussier during an autopsy in 1770. The incidence of this condition ranges from 0.01% to 0.98%, with up to 80% of cases remaining asymptomatic until incidental discovery.^1^ Typically, true splenic artery aneurysms are more prevalent in women and are diagnosed using computer tomography (CT).^2^ These aneurysms rarely exceed 3 cm in size, with only a few reported cases of giant splenic artery aneurysms (greater than 10 cm). Delayed diagnosis and treatment can lead to severe complications, as the lifetime risk of rupture ranges from 2-10% for small aneurysms to as high as 28% for giant aneurysms.^3^ In the event of rupture, mortality rates can be as high as 25-75%. Consequently, surgical intervention remains the sole viable treatment option.

In our article, we present a clinical case involving successful removal of a giant splenic artery aneurysm in a 55-year-old female patient who was admitted to the surgical clinic of Botkin Hospital in Moscow, Russian Federation.

## CASE DESCRIPTION

Patient X, a 55-year-old-woman, visited her local health clinic complaining of pronounced weakness, discomfort, and pain in her left subcostal area that had persisted for four months. From anamnesis it was found that the patient was not a smoker, did not suffer from hypertension, and had not had multiple pregnancies. No liver pathology was observed. There was no history suggestive of pancreatitis, peptic ulcer disease, abdominal trauma, hepatobiliary disease, previous surgery, or any other significant illness. On examination, she was comfortable with blood pressure at 100/70 and pulse rate at 82 per min−1. Pallor was absent. A well-defined, non-tender, pulsatile intra abdominal lump was palpable in the left hypochondrium, left lumbar, and epigastric region, which was oval in shape and 12 cm × 10 cm in size.

**Figure 1:**
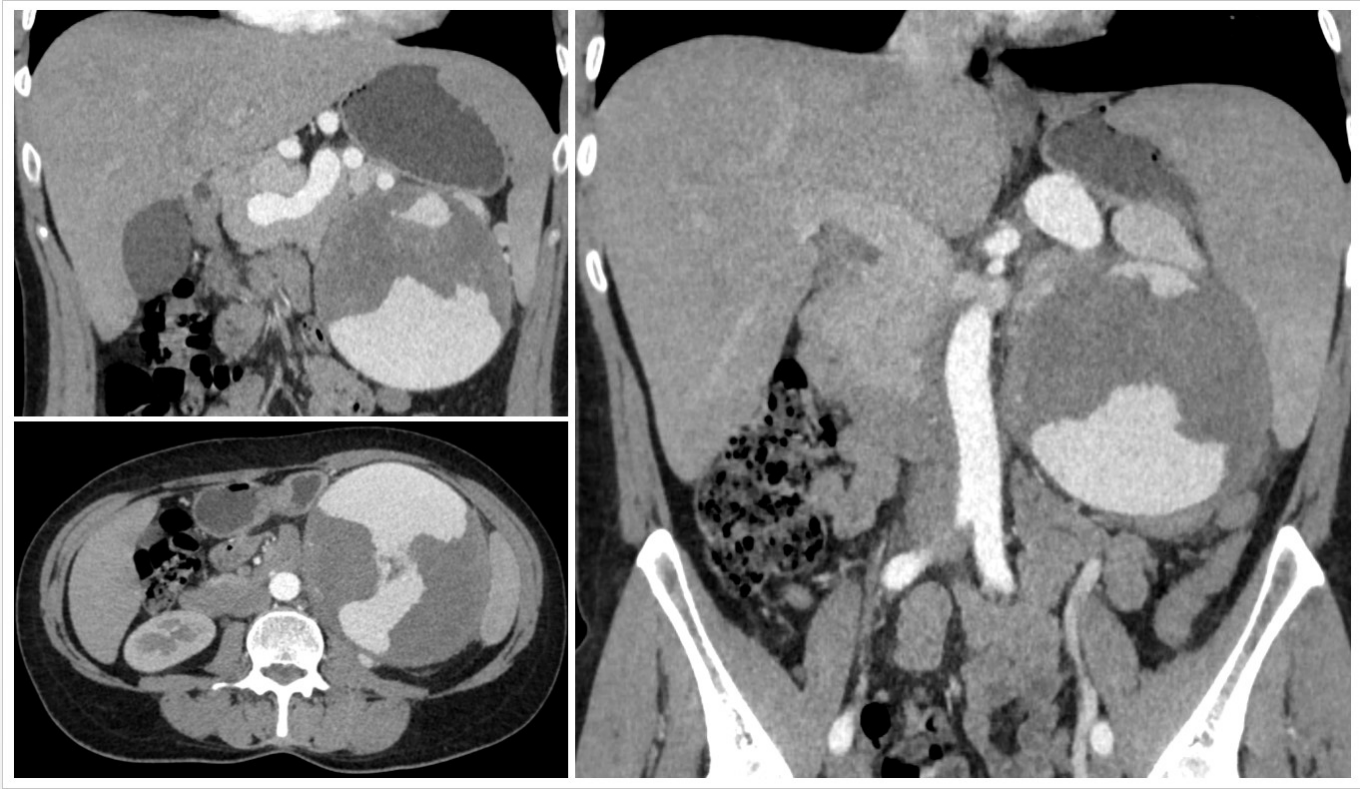
Patient X. Computed tomography of the abdomen with intravenous contrast. Giant splenic artery aneurysm (12x10x12 cm).

An abdominal CT scan was performed, revealing a 120x123 mm splenic artery aneurysm with irregularly shaped hypoechogenic thrombotic masses, occupying two-thirds of the lumen volume (Figure 1). As a result of this diagnosis, the patient was urgently admitted to the surgical clinic at Botkin Hospital and later transferred to the hepatopancreatobiliary surgery department.

Ultrasound investigation of the spleen in the projection of the splenic hilum revealed a globular aneurysmal dilatation of the arterial vessel, associated with the splenic artery and measuring up to 125x100 mm. The lumen contained hypoechogenic aneurysmal thrombotic masses and occupied two-thirds of the lumen volume. No fluid accumulations were observed in the vicinity of this splenic artery aneurysm. No other pathology was detected by X-ray and ultrasound examination. According to the laboratory investigation, the patient had moderate iron deficiency anemia (Hb - 96.1 g/l, Fe - 1.9 µmol/l).

The patient underwent a comprehensive preoperative examination, which included esophagogastroduodenoscopy and colonoscopy, revealing no significant organic pathologies. During EchoCG: aortic wall, aortic valve leaflets were compact, there was 1st degree mitral valve prolapse, cavities were not dilated, systolic function was good.

The patient subjectively noted an improvement in health status against the background of conservative treatment (antispasmodic, iron supplementation).

However, due to the high risk of rupture associated with giant splenic artery aneurysms and the ineffectiveness of endovascular treatment due to the artery’s size and tortuous course, the patient was prepared for elective surgery.

**Figure 2:**
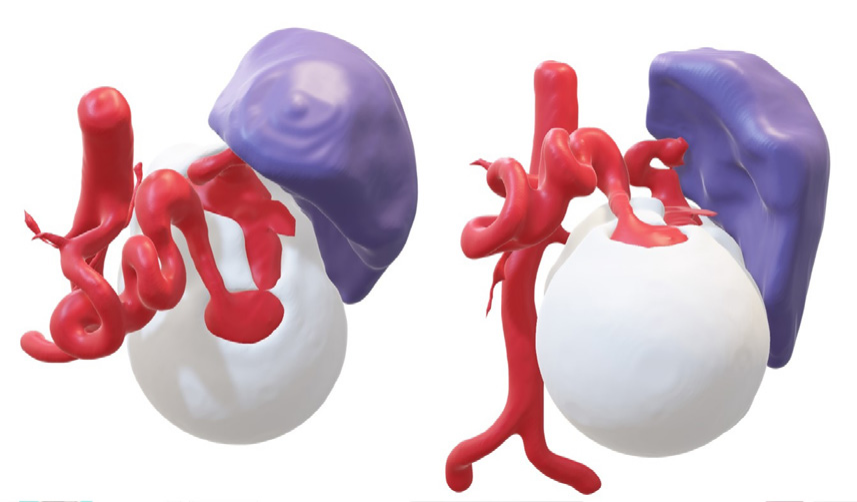
Patient X. 3D reconstruction of a giant splenic artery aneurysm.

Preoperative planning of surgical treatment was based on three-dimensional modeling of the anatomy of the splenic artery using multiphase abdominal computed tomography (Figure 2). In this patient, the splenic artery had a tortuous course, which was also a contraindication for endovascular treatment.

**Figure 3:**
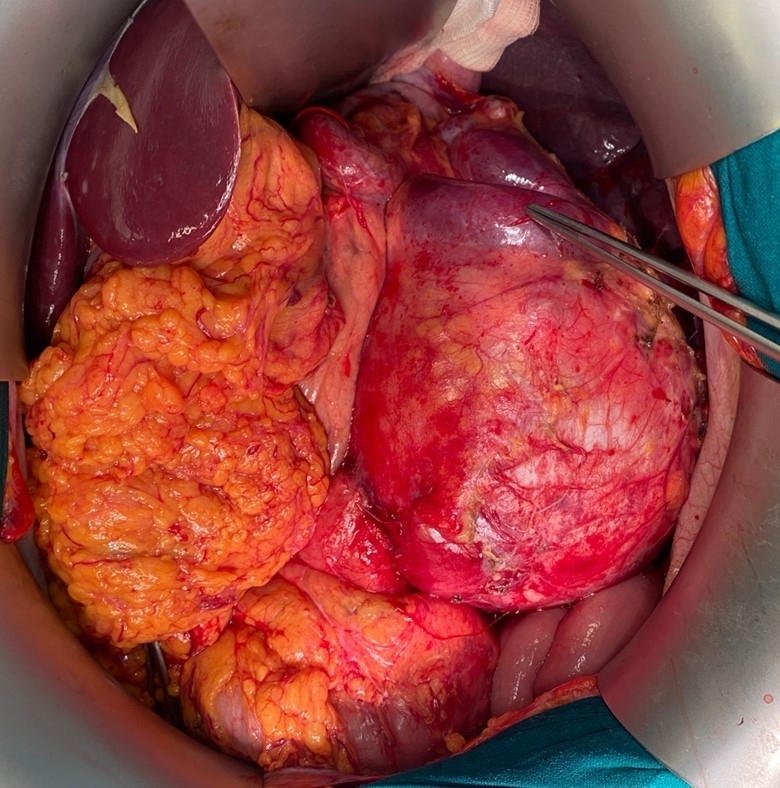
Intraoperative image.

The patient underwent open surgical repair. Surgical intervention was performed in the form of aneurysm resection along with splenectomy and distal resection of the pancreas. After a midline laparotomy and abdominal cavity revision, a large pulsating mass was detected in the retroperitoneal space originating from the splenic artery, 12x12 cm in size, located in the body/tail of the pancreas, and occupying the upper abdominal floor, (Figure 3). Given the pronounced adhesions in the abdominal cavity, in the area of the spleen, splenic flexure of the colon, and pancreas, adhesiolysis was performed with technical difficulties. The aneurysm was tightly connected to the splenic angle of the transverse colon, the tail of the pancreas, and the spleen. Using LigaSure and bipolar coagulation, the splenic angle of the transverse colon was mobilized. Subsequently, the splenic artery and vein were isolated, and a vascular clamp was placed on the splenic artery, resulting in cessation of the pulsations in the mass, confirming the aneurysm’s origin from the splenic artery. The splenic artery and vein were clipped with Hem-o-Lock clips and crossed and the splenic artery stump was sutured. The tail of the pancreas was mobilized and resected using LigaSure and bipolar coagulation. Distal pancreatic resection was performed with splenectomy and resection of the splenic artery aneurysm. The organ complex was removed and sent for histological examination. The pancreatic stump was sutured with Z-shaped sutures. A hemostatic sponge was placed on the pancreatic stump. Two drains were installed in the cavity of the removed organ complex, to the pancreatic stump, and were removed through the contra-aperture in the left mesogastric region. The approximate blood loss was 200 ml.

**Figure 4:**
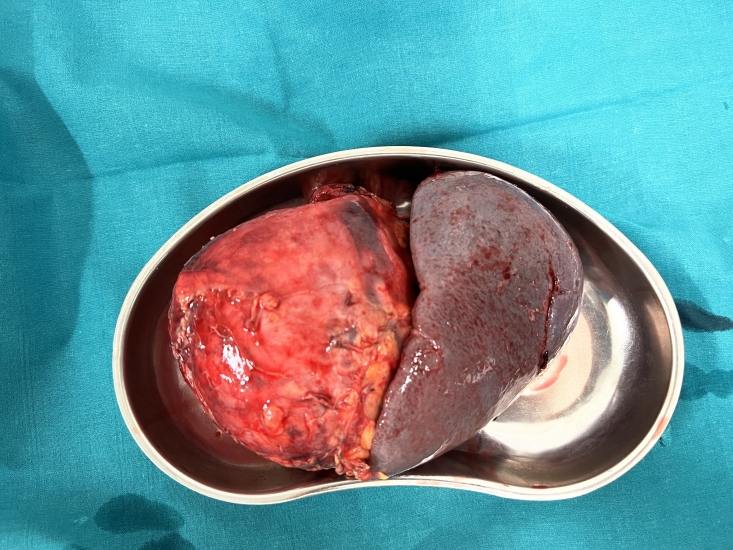
Macrospecimen of splenic artery aneurysm.

On macroscopic examination, the aneurysm was 12x10x12 cm in size and of dense consistency, fused to 13 cm of the spleen and up to 1 cm of the tail of the pancreas (Figure 4).

**Figure 5:**
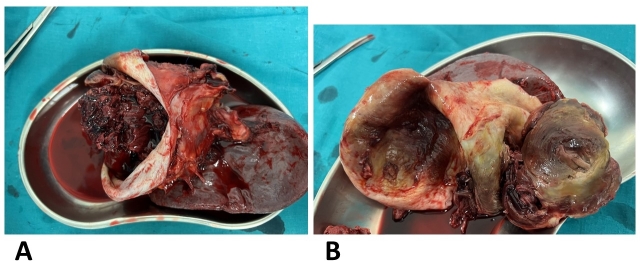
Macrospecimen of an aneurysm of the splenic artery in section. (A) The contents of the aneurysm; (B) aneurysm cavity.

Upon dissecting the aneurysm, its interior surface displayed a yellowish appearance, with areas showing calcium salt deposition (dystrophic calcification) and the aneurysm cavity contained long-standing thrombotic masses (Figure 5).

**Figure 6:**
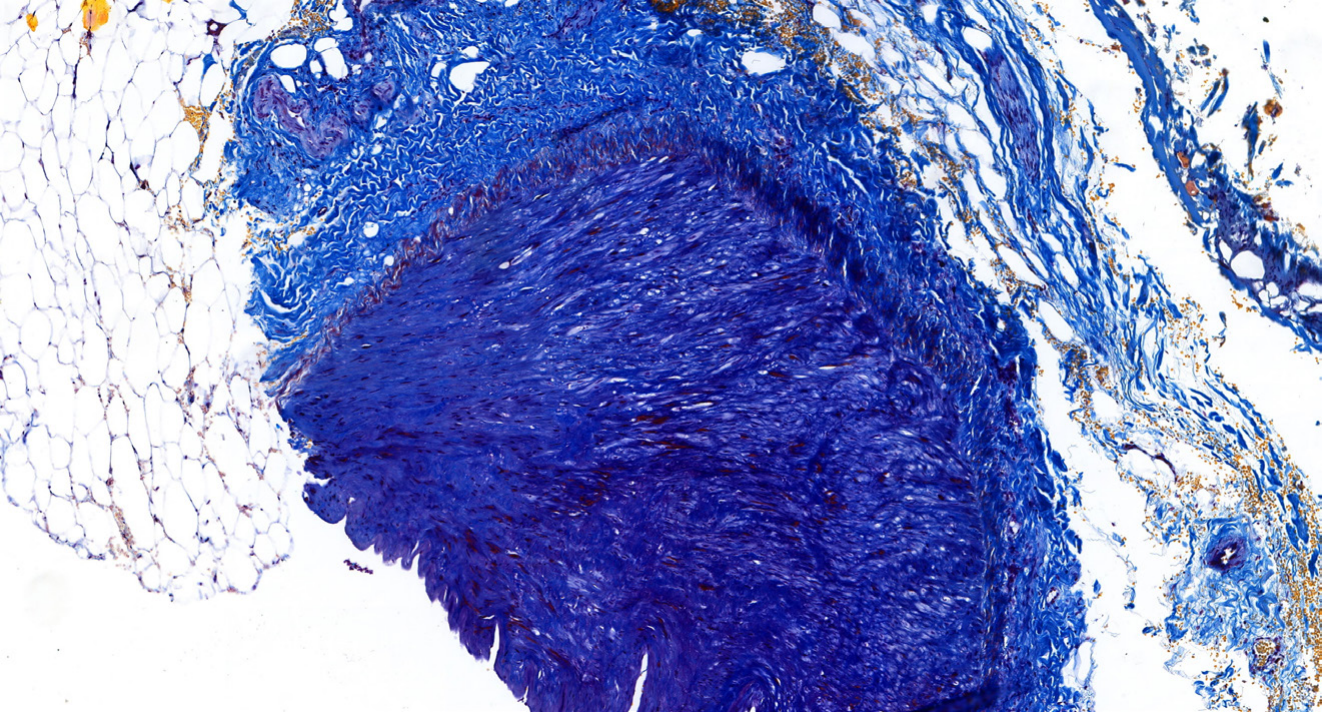
Histological image of a splenic artery aneurysm. Atherosclerotic lesions.

**Figure 7:**
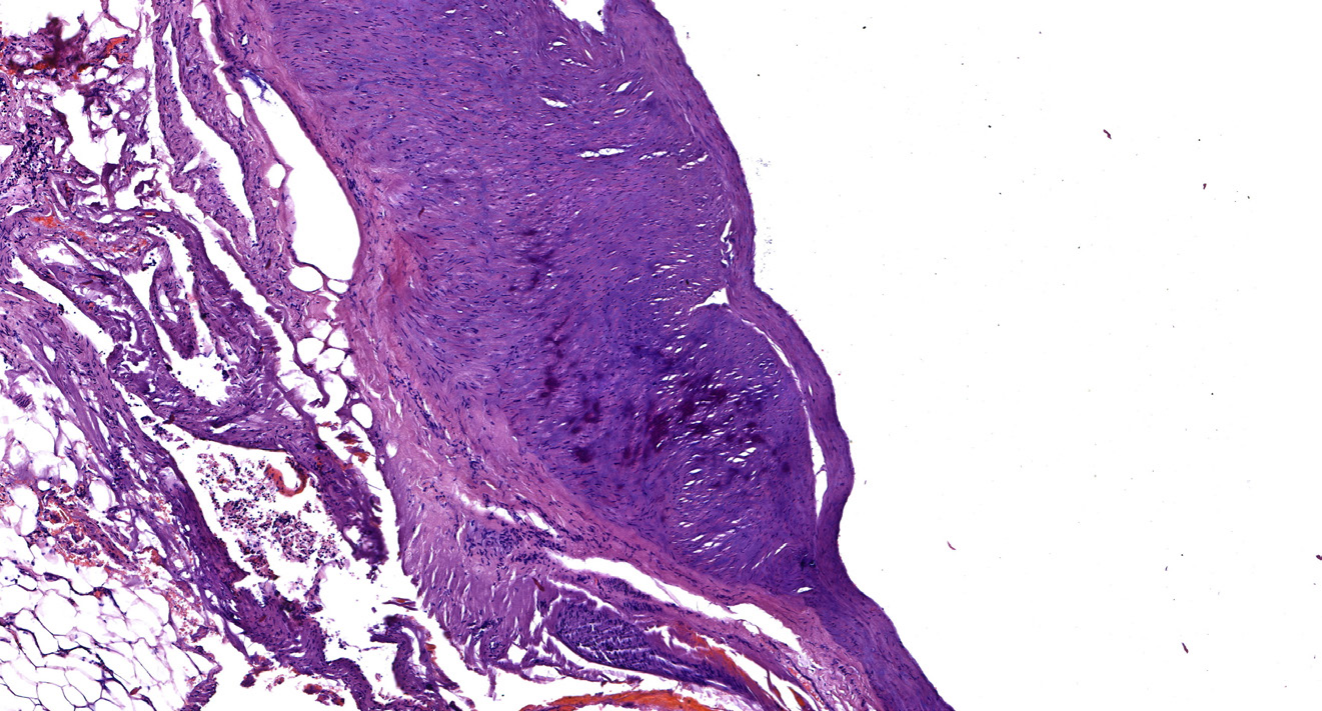
Histological image of a splenic artery aneurysm. Connective tissue dysplasia.

Histopathology showed the aneurysm involved all three layers of the vessel wall, suggestive of a true aneurysm. Microscopic examination revealed a sharply dilated aneurysm, with absent endothelium in some areas. The inner sheath was thickened in places, while the middle membrane exhibited muscular layers that were markedly thinned and elongated. There was a substantial increase in the number of elastic fibers, forming compact strands that were torn in some places. The intermuscular connective tissue showed increased density and, in some areas, penetrated between the muscle fibers, separating them (Figures 6 & 7).

In the postoperative period, the patient received comprehensive antibacterial, anticoagulant, antispasmodic, and symptomatic therapy, with dynamic monitoring and regular checks of laboratory parameters. Fortunately, the postoperative phase was without complications, and the patient was discharged in satisfactory condition under the care of local healthcare providers. The length of postoperative care was two weeks. No adverse events occurred during 6 months of follow-up.

## DISCUSSIONS

To date, consensus on the management and treatment of patients with true splenic artery aneurysms remains elusive. However, surgical treatment is universally indicated for all symptomatic splenic artery aneurysms, regardless of size. Treatment is also recommended in asymptomatic patients with lesions ≥2 cm in size, pregnant or fertile patients with portal hypertension, or liver transplant candidates.^4^ Giant splenic artery aneurysms (greater than 10 cm) are infrequent, but have a significantly higher risk of rupture, necessitating surgical intervention.

The most common treatment options for splenic artery aneurysms are open, endovascular (embolization with coils or stent grafts), and laparoscopic interventions. The choice of method depends on the age and general condition of the patient and anatomical features of the aneurysm.

Open interventions are reserved for cases with unstable hemodynamics or aneurysm rupture and are considered the “gold standard” for treatment of large splenic artery aneurysms.^5,6^ Several clinical cases of laparotomy with splenic artery aneurysm resection due to the giant size of the aneurysm have been documented.

Endovascular embolization of a splenic artery aneurysm is performed when the aneurysm has a narrow neck with a straight course, while stent grafting is used for an aneurysm with a wide neck.^7^ This procedure is technically complex and expensive, which prevents this treatment option from being widely used. Postoperative “double” antiplatelet therapy may adversely affect quality of life, especially for young patients, and complications like recanalization and post-embolization syndrome can occur.^8^

Laparoscopic clipping of splenic artery aneurysm branches is feasible regardless of the aneurysm’s location.^9^ Laparoscopic splenectomy is recommended for multiple splenic artery aneurysms or when the aneurysm is densely fused with the pancreatic tail in the gate region. Laparoscopic resection of a splenic artery aneurysm serves as an alternative to open interventions. However, this technique is not recommended for giant aneurysms with lesions and tight fusion with the surrounding tissue. No descriptions of laparoscopic resection for giant splenic artery aneurysms were found in the literature review.

Several cases of giant splenic artery aneurysms were described and the authors resorted to different surgical techniques.

In giant aneurysms, when simple aneurysmectomy is not possible because of tight adhesions, surgical treatment options include aneurysmectomy with splenectomy, splenic artery ligation with/without aneurysmectomy and, if necessary, distal splenic artery resection.^10,11^

In our case, distal pancreatic resection with splenectomy and resection of splenic artery aneurysm was performed for a giant splenic artery aneurysm directly involving a long segment of the tail of the pancreas, where its mobilization would have caused damage to the pancreatic parenchyma, with subsequent development of acute destructive pancreatitis. Despite the spleen remaining well vascularized, its removal was necessary due to a considerable capsular tear.

Kalipatnapu et al.^12^ performed splenic artery ligation proximal to the aneurysm entry point, evacuated the aneurysm contents, and subsequently ligated the distal branch of the splenic artery aneurysm; their patient was discharged in satisfactory condition.^12^

Panzera et al.^13^ described a clinical case of aneurysm resection with splenectomy and an additional distal splenic artery resection due to splenic artery aneurysm invasion and adhesion to the pancreatic tail.^13^

Endovascular treatment is performed in the presence of strict indications and is mainly used for small and non-functioning splenic artery aneurysms. Wernheden et al.^14^ found it impossible to embolize an aneurysm with coils due to its large size and could not visualize the distal branch of the aneurysm. However, they were able to embolize the proximal branch of the splenic artery and collateral vessels lateral to the aneurysm to prevent reverse perfusion.^14^ Endovascular treatment was preferred in this case due to the patient’s advanced age and the suitability of the aneurysm for endovascular embolization.

In addition, Herskowitz et al.^15^ presented a case in which successful treatment of a giant splenic artery aneurysm was achieved using proximal embolization alone.^15^

Therefore, timely diagnosis of splenic artery aneurysm with determination of its size and anatomic peculiarities is crucial to guide selection of the appropriate surgical intervention, ultimately ensuring favorable prognosis for the patient.

Despite the successful surgical management of this giant splenic artery aneurysm, it is crucial to acknowledge certain limitations to our study design. The follow-up period in this study extended up to two weeks post-surgery, during which the patient was discharged without any immediate complications. However, the relatively short follow-up period may not capture potential longer-term developments or complications that could arise in these patients. To address this limitation, we strongly recommend that patients be scheduled for follow-up CT imaging beyond the six-month mark to track the evolution of their condition. While our initial findings are promising, a more extended follow-up period will provide a more comprehensive understanding of the long-term outcomes of surgical intervention in patients with giant splenic artery aneurysms and we aim to incorporate this into our future research efforts.

## CONCLUSIONS

In conclusion, giant splenic artery aneurysms represent a rare group of diseases. Preoperative diagnosis of such patients includes computed tomography with intravenous contrast and 3D reconstruction of the splenic artery to determine the most appropriate surgical approach. The clinical case presented here is noteworthy due to the aneurysm’s significant size, the intricacies of the diagnostic process, the extent of the surgical intervention chosen, and the subsequent successful rehabilitation and complete recovery of the patient.
